# Recent Advances in Cellulose-Based Hydrogels: Food Applications

**DOI:** 10.3390/foods12020350

**Published:** 2023-01-11

**Authors:** Pinku Chandra Nath, Shubhankar Debnath, Minaxi Sharma, Kandi Sridhar, Prakash Kumar Nayak, Baskaran Stephen Inbaraj

**Affiliations:** 1 Department of Bio Engineering, National Institute of Technology Agartala, Jirania 799046, India; 2 Haute Ecole Provinciale de Hainaut-Condorcet, 7800 Ath, Belgium; 3 Department of Food Technology, Karpagam Academy of Higher Education, Coimbatore 641021, India; 4 Department of Food Engineering and Technology, Central Institute of Technology Kokrajhar, Kokrajhar 783370, India; 5 Department of Food Science, Fu Jen Catholic University, New Taipei City 242062, Taiwan

**Keywords:** cellulose-based hydrogels (CBHs), food industry, functional food, biodegradation, food packaging

## Abstract

In the past couple of years, cellulose has attracted a significant amount of attention and research interest due to the fact that it is the most abundant and renewable source of hydrogels. With increasing environmental issues and an emerging demand, researchers around the world are focusing on naturally produced hydrogels in particular due to their biocompatibility, biodegradability, and abundance. Hydrogels are three-dimensional (3D) networks created by chemically or physically crosslinking linear (or branching) hydrophilic polymer molecules. Hydrogels have a high capacity to absorb water and biological fluids. Although hydrogels have been widely used in food applications, the majority of them are not biodegradable. Because of their functional characteristics, cellulose-based hydrogels (CBHs) are currently utilized as an important factor for different aspects in the food industry. Cellulose-based hydrogels have been extensively studied in the fields of food packaging, functional food, food safety, and drug delivery due to their structural interchangeability and stimuli-responsive properties. This article addresses the sources of CBHs, types of cellulose, and preparation methods of the hydrogel as well as the most recent developments and uses of cellulose-based hydrogels in the food processing sector. In addition, information regarding the improvement of edible and functional CBHs was discussed, along with potential research opportunities and possibilities. Finally, CBHs could be effectively used in the industry of food processing for the aforementioned reasons.

## 1. Introduction

Gels are networks of polymers that are swollen by a large amount of solvent and have three dimensions. Hydrogels are structures that are mostly made of biopolymers and/or polyelectrolytes and retain a great amount of water [1]. According to the source, there are two types of hydrogels: those that are made from natural polymers and those that are made from synthetic polymers. Based on cross-linking, hydrogels can be put into two groups: chemical gels and physical gels. Physical gels are made when molecules stick together on their own through ionic or hydrogen bonds. Chemical gels are made when molecules stick together through covalent bonds [2]. The first hydrogels were described by Wichterle and Lim [3]. Notably, hydrogels have several potential uses in the fields of food, agriculture, water purification and biomaterials, etc. Recently, researchers have actively contributed to the development of innovative hydrogels for implementations such as recyclable materials for drug delivery [4,5], tissue engineering [6,7,8], sensors [9,10], contact lenses [11,12], and purification [13], etc. There have been reports of synthetic polymer-based hydrogels, such as those made by crosslinking *polyethylene glycol* [14], *polyvinyl alcohol* [15], *polyamidoamine* [16], poly(N-isopropylacrylamide) [17], polyacrylamide [18], polyacrylic acid [19], and their co-polymers [20]. Synthetic hydrogels, such as PEG-based hydrogels, have benefits over natural hydrogels, for example the potential for photo-polymerization, adjustable mechanical characteristics, and simple operation of scaffold architecture and chemical characteristics. However, due to their bio-inert nature, PEG hydrogels are unable to provide an optimal environment in which cells can practice adhesion and tissue engineering [21]. A portion of polysaccharides are comparable to PEG in terms of biocompatibility; they have low protein and cell adhesion, and can biodegrade into nontoxic, easily absorbed compounds [22]. Utilizing hyaluronate [23], alginate [24], starch [25], gelatin, cellulose [26], chitosan, and their derivatives [27,28,29], numerous hydrogels from biopolymers have already been created, demonstrating potential application in the biomaterials field due to its safety, wettability, and bio-compatibility. Cellulose is the basic structural component of plants, and since plants are the first link in the food chain (which defines the feeding relationships between all living creatures), cellulose is a crucial substance. The most prevalent biopolymer found in nature [30,31]. The primary component of numerous natural fibers, such as cotton and higher plants, is cellulose [32,33]. It consists of lengthy chains of anhydro-D-glucopyranose units (AGU), with the exception of the terminal ends, each cellulose molecule having three hydroxyl groups per AGU [32].

Recent studies have shown that cellulose-based hydrogels can be used as freshness metrics. These hydrogels can detect modifications in pH, chemical breakdown, and the development of microorganisms in food based on the generation of metabolic pathways in the food. In addition, cellulose-based hydrogels are usually employed for food packaging [34], which improves the freshness and safety of perishable goods, especially those that are not pre-packaged. Cellulose gives the packaging material barrier properties by slowing the diffusion of liquids, gases, and solids. Furthermore, CBHs serve as a method of regulating humidity, particularly for damp food sources such as ready-to-serve products and items that absorb and release water (powdered form food), by lowering water activity, preventing microbe growth, and reducing the degree to which dry, crunchy foods are softened (biscuits and fried potatoes) [35]. Similarly, flavor or volatile substances are important and complex metrics in foods that are unsteady due to environmental factors. CBHs prevent the deterioration of flavor compounds [36]. Studies have shown evidence of the production of cellulose-based gels and their applications over the years [27,28,29]. More recently, cellulose-based hydrogels are being investigated for a wide variety of food applications. Therefore, a systematic review dealing with the production of cellulose gel, their mechanisms, and their extensive food applications should provide substantial knowledge on gel production and their novel food applications. For instance, Thivya et al. [37] reviewed the recent studies on cellulose-based hydrogels being limited to food industry applications. Thus, a comprehensive review of the latest trends in CBHs production, mechanism, characteristics, and food applications is necessary. Hence, this review primarily focuses on the possibilities and advancements of cellulose-based hydrogels in the food processing sector. Furthermore, a clear and concise explanation of the fundamental ideas, classifications, and preparation techniques pertaining to hydrogels is also presented.

## 2. Sources of Cellulose-Based Hydrogels Production

**Figure 1:**
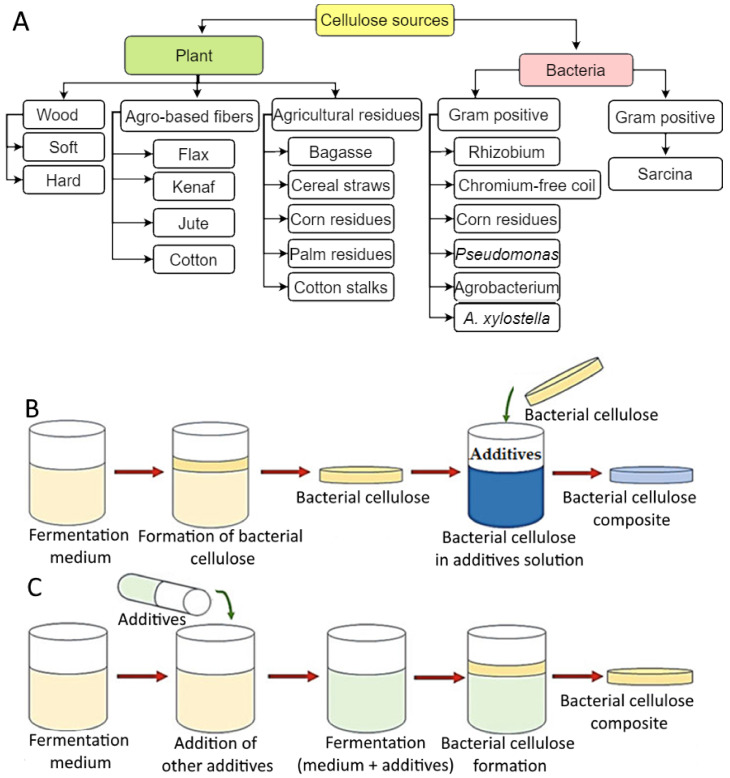
Cellulose sources for hydrogel production (**A**), and modification of bacterial cellulose using (**B**) ex situ and (**C**) in situ methods. Figure 1A is adapted from Chen et al. [41] and is an open access article (Copyright © 2022 by authors) distributed under the terms and conditions of the Creative Commons Attribution (CC BY) license, while Figure 1 (B and C) is adapted with permission (Copyright © 2019 Springer Nature Switzerland AG, Cham, Switzerland) from Sabbagh et al. [42].

Cellulose is the most abundant natural glucose polymer, and it is abundantly generated from various agricultural residues (Figure 1A). Cellulose has superior heat stability during excessive temperatures and acts as a UV ray protector. Because of their mechanical strength, biocompatibility, and environmental sustainability, cellulose and its derivatives have received a lot of attention inside the international market, especially regarding food, biomedicine, and fabric applications.

Bacterial cellulose (BC) or microbial cellulose is chemically identical to plant cellulose (PC), but their physical structures and macromolecular structures are distinct. The insolubility in water and other solvents as well as the high crystallinity of the cellulose in both PC and BC are a result of the 1,4-β-glucosidic bonds between glucose units [38]. This quantity exceeds 60% for BC and falls between 40% and 60% for PC [39]. The nanosized fibers are the outcome of BC biosynthesis and are approximately two orders smaller than PC fibers. Therefore, BC cellulose has an ultrafine and distinctive fiber network with greater flexibility and greater water retention than PC [39]. In addition, BC is composed solely of PC, which is unrelated to the remaining biogenic components, such as pectin and lignin [40]. Therefore, anytime BC is utilized by bacteria, PC must be refined and reformed. In the meantime, further modification of BC might be performed ex situ or in situ (Figure 1B,C), in order to generate desirable form and properties utilizing various types of additives, such as conductive polyaniline nanoparticles.

## 3. Different Types of Hydrogels

**Figure 2:**
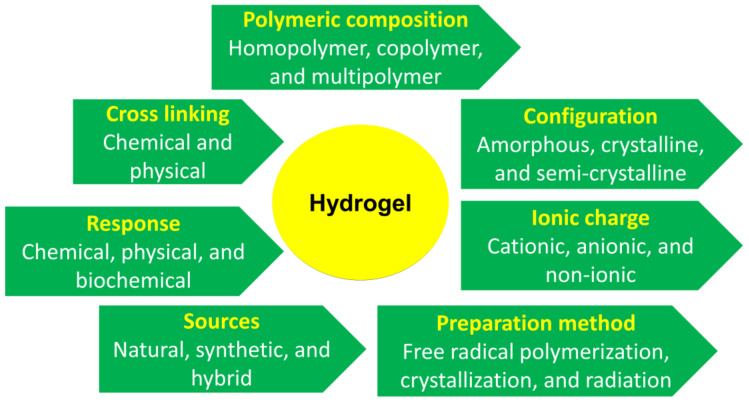
Classification of hydrogels. Figure 2 is adapted with permission (Copyright © 2022 Elsevier B.V., Amsterdam, The Netherlands) from Hasan et al. [44].

Hydrogels are classified according to their physical parameters, nature of swelling, preparation methods, ionic charges, sources, rate of degradability, and observed nature of crosslinking [43] as shown in Figure 2.

In physical gels, the crosslinking process is physical in nature. Typically, this is accomplished through physical processes including hydrophobic association, chain aggregation, crystallization, polymer chain complexion, and hydrogen bonding. In contrast, a chemical process, namely chemical covalent crosslinking (simultaneously or post polymerization), is used to prepare a chemical hydrogel. As a result of conformational changes, physical hydrogels are reversible, whereas chemical hydrogels are permanent and irreversible.

Due to electrostatic interaction, the combination of physically and chemically cross-linked hydrogels results in the formation of dual-network hydrogels. Recently, it has been used to address the limitations of using only chemical or physical hydrogels with a high liquid uptake capacity over a wide pH range and greater sensitivity to pH changes than chemical hydrogels. Cong et al. [45] and Yalpani [46] recently reported another graphene-polymer composite material that forms a dual-network with exceptional mechanical properties and self-healing abilities.

## 4. Crosslinking in Hydrogels

Different methodological approaches are used to make hydrogels for different purposes. Due to this, hydrogel characteristics such as the highest mechanical performance, chemical characteristics, density, degradability, biological response, and response to the surrounding have been different [47]. Table 1 shows the materials, methods, and applications employed in the preparation of hydrogels.

### 4.1. Physical Crosslinking

**Figure 3:**
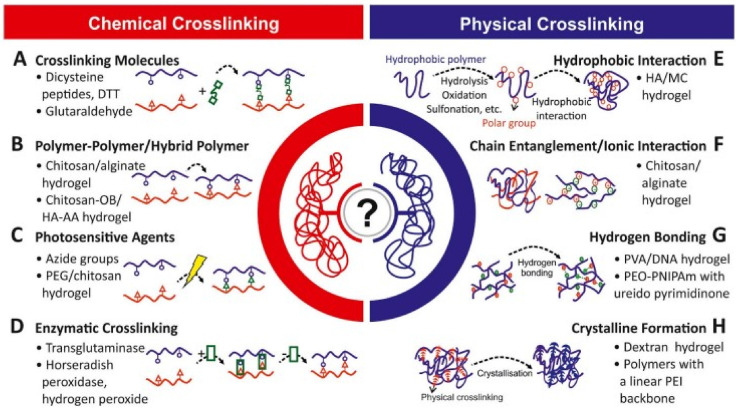
Different crosslinking methods used to produce hydrogels. Figure 3 is adapted from George et al. [78] and is an open access article (Copyright © 2019 by authors) distributed under the terms and conditions of the Creative Commons Attribution (CC BY) license.

Physical crosslinking has been an intriguing technique for preparing hydrogels due to the nature of crosslinking agents used. In addition, physical crosslinking does not disrupt living organisms, though it does enhance hydrogel formation. There are a wide variety of incarnations for each method of physical crosslinking. The subsequent procedures are used to create physically cross-linked hydrogels (Figure 3).

#### 4.1.1. Crosslinking by Radical Polymerization

The amount of the cross-linker can determine how much the hydrogel swells, which is one of the characteristics that distinguish hydrogels. Furthermore, the inclusion of a cross-linker with specific characteristics can result in the creation of materials that are responsive to external stimuli. In addition to obtaining chemically cross-linked hydrogels through the radical polymerization of mixtures of vinyl-monomers, radical polymerization of water-soluble polymers that have been modified with polymerizable groups is another method for obtaining chemically cross-linked hydrogels. In order to create hydrogels with this technique, a range of water-soluble polymers, including synthetic, semi-synthetic, and natural varieties, have been used.

#### 4.1.2. Crosslinking by Ionic Interactions

Hydrogels can be classified by the types of interactions between their hydrophilic backbones and ions (anionic, cationic, and amphoteric). Moreover, the inclusion of di- or tri-valent counterions caused ionic contact between the polymers, leading to the formation of a hydrogel system. The basic idea behind this technique is to cause a polyelectrolyte solution to gel by adding multivalent ions with opposite charges. Anionic hydrogel is formed from polymers with a negative charge, whereas cationic hydrogel is produced from polymers with a positive charge [79]. Positive and negative ions are present in equal numbers in neutral hydrogel [80]. The swelling impact of the aqueous medium determines the degree of ionic chain dissociation in cationic hydrogels. Similarly, in acidic environments with low pH, cationic hydrogels disintegrate, whereas anionic hydrogels swell better. At a particular pH, amphoteric hydrogels balance both positive and negative charges. Changes in the pH of the solution can have an effect on the ionic characteristics of these hydrogels [81]. Non-ionic hydrogels swell in aqueous solution in the absence of crosslinkers. By replacing a hydrophilic backbone with pendant hydrophobic groups and modifying the temperature of the aqueous solutions, the hydrophobic groups in hydrogels are modified, resulting in a formulation that balances the interactions of hydrophilic and hydrophobic groups.

#### 4.1.3. Crosslinking by Host-Guest Interactions

Development of supramolecular polymer hydrogels based on the host-guest assembly method is of particular interest because these substances have unique non-covalent and dynamic binding motifs that can be targeted by external stimuli and easily tuned by changing the crosslinks’ architecture and density. Numerous studies on cyclodextrin (CD) inclusion complex-based supramolecular polymer hydrogels have been published. CD chemically linked on cellulose chains [82], cellulose derivatives [83], or CNCs surfaces [84] serve as the host when creating supramolecular CBHs. The guest polymers can be adamantane (AD) moities [85], azoben zene [84], pluronic polymers [84], or azoben polymers [83].

Cucurbit [8]uril (CB [8]) is another macrocyclic molecule that can accommodate up to two aromatic guest molecules at the same time inside its cavity to form either 1:2 CB [8]•(guest)2 homoternary complexes with monocationic guests or 1:1:1 heteroternary complexes with both a dicationic and a neutral guest [86,87], which is the best choice as a crosslinker when making polymer gels. Naphthyl-functionalized CNC (CNC-g-P(DMAEMA-r-NpMA)) [88] and methyl-viologen-functionalized PVA were held together by CB [8] through supramolecular crosslinks created by dynamic host-guest interactions as well as selective and simultaneous binding of naphthyl and methyl viologen moieties. These kinds of methods can be used to make advanced, dynamic materials from renewable sources. Using single emulsion droplet microfluidics and macrocyclic host–guest complexation between the macrocyclic host cucurbit [8] uril (CB [8]) and the guest of anthracene-functionalized hydroxyethyl cellulose (Ant-HEC) polymers, supramolecular hydrogel microcapsules have been made [89]. The head-to-tail arrangement of two anthracene moieties within the cavity of CB [8] allows multiple non-covalent crosslinks to form between adjacent anthracene-functionalized polymer chains, creating supramolecular polymer hydrogel skins at the water-in-oil interface of single micro-droplets.

#### 4.1.4. Crosslinking by Crystallization

PVA [poly(vinyl alcohol)] is a polymer that is water-soluble. PVA aqueous solutions kept at ambient temperature transform into a weak gel over time. Interestingly, a highly elastic gel is created when the aqueous solutions of this polymer undergo a freeze–thaw cycle [90]. The gel’s characteristics depend on the molecular weight of PVA, the concentration of PVA in water, the freezing temperature and time, and the number of freezing cycles. The formation of PVA crystallites that serve as physical crosslinking sites in the network is attributed to gel formation. Gels prepared under ambient conditions are steady at 37 °C for six months [91]. Hydrogels with physical cross-links are typically made from multiblock or graft copolymers. The latter can have either a hydrophobic chain with a water-soluble graft attached to it or a polymer backbone that is soluble in water but to which hydrophobic units have been attached. H-bonding [45], polymerization in suspension [92], chemical reaction of identical groups caused by irradiation [93] and protein crosslinking [94] have also been reported as methods of crosslinking, but they all require the use of a crosslinking agent, which can be toxic and raise questions about the gel’s durability. For this reason, physically cross-linked hydrogels are now available and can be prepared via a variety of crosslinking methods such as hydrophobic interaction, H-bonding, protein interaction, and crystallization of ionic interactions [95].

#### 4.1.5. Freeze–thaw Process, Hydrogen-Bonding, and Complex Coacervation

Simple mixing of polycation and polyanion produces hydrogels produced by complicated coacervation. Due to their opposing charges, the polymers adhere to one another and create soluble and insoluble complexes depending on the concentration and pH of the solutions. The freeze–thaw process causes the formation of microcrystals inside the structure of the substance as a consequence of repeated cycles of freezing and thawing. These hydrogels’ linked hydrogen bonding networks provide them spongier, porous, rubberier, and more elastic characteristics [96]. Currently, such hydrogels are frequently used in the biotechnology fields, especially for the immobilization of entire cells and molecules (protein, peptides) [97]. A non-covalent bonding method called “H-bonding” is used to create physically crosslinked hydrogels. The sodium ion in carboxymethyl cellulose (CMC) replaces the hydrogen ion as it dissolves in 0.1 M HCL [98]. The elasticity of the hydrogel created by H-bonding lowers the solubility of CMC in water.

#### 4.1.6. Maturation

Hydrogels with precise molecular structure measurements are made using the maturation process, which involves heat-induced aggregation. Arabic-gum-cellulose is the best example of heat-induced hydrogels. Gum Arabic (Acacia gums) is a carbohydrate whose structural protein content ranges from 2–3% [99]. Heating distinct protein groups, such as arabinogalactan protein, arabinogalactan, and glycoprotein with differing molecular weights, causes the accumulation of proteinaceous groups [100]. In order to enhance their ability to bind water and mechanical strength, cellulose and gum-Arabic hydrogels generate cross-linked networks by converting low molecular weight protein groups into high molecular weight protein groups.

### 4.2. Chemical Crosslinking

Chemical crosslinking in hydrogels typically focuses on the linkages between the polymer and the crosslinking agent. A certain functional group of the crosslinking agent determines the hydrogel’s properties, particularly its mechanical strength. Polymers having hydroxyl groups can be crosslinked with glutaraldehyde under extreme conditions: low pH, high temperature, and the inclusion of methanol as a quencher [91]. Crosslinking polysaccharides with 1,6-hexamethylene diisocyanate, divinyl sulfone, and other compounds can result in the formation of hydrogels [101]. The goal of these chemicals is to ensure that a specific polymer or functional group creates a network of interconnecting linkages to produce hydrogels [102].

#### 4.2.1. Citric Acid (CA)

Citric acid has been employed as a crosslinking agent because it is cheap, nontoxic, water-loving, and a natural organic with 3–OH groups that can form a network in most hydrogel preparations. It has been shown that citric acid can make strong hydrogen-bonds that make water expand and stay stable at high temperatures. It has an additional binding site and contains hydrogen-bonds, which help maintain hydrophilicily balance [103,104,105]. Food contact materials, water softeners, anticoagulants, antiviral tissues, and cleaning supplies all feature citric acid, also known as food-additives [106,107]. CA also makes the hydrogel network stronger by improving the tensile properties, heat resistance, and impermeability better [108,109,110]. CA is widely recognized as an effective crosslinking agent that is utilized a lot to make cellulose hydrogels, which improves their properties. Additionally, CA is an organic substance that has been authorized by the FDA (Food & Drug Administration) for use as a secure crosslinking agent or to be consumed by the body. Researchers have found that CA is the easiest cross-linker to utilize at ambient temperature to make hydrogels [111,112].

The esterification-crosslinking process was used to generate β-cyclodextrin–carboxymethylcellulose (βCD-CMC) hydrogel films for the controlled release of ketoconazole (model drug) [113]. The active βCD content, carboxyl content, and degree of crosslinking in the hydrogel films increased as the concentration of βCD in the feed increased; however, at high concentrations, the carboxyl content and interpolymer crosslinking decreased [114]. The presence of βCD in the hydrogel films assisted in minimizing the drug’s burst release. The βCD-CMC hydrogel films were able to control the drug release over an extended period of time [113]. The hemolytic assay revealed the biocompatibility of the hydrogel films. Therefore, βCD-CMC hydrogel films are more effective than βCD-HPMC hydrogel films; although their efficacy for drug delivery cannot be proven until cytocompatibility and in vivo tests have been conducted [113].

#### 4.2.2. Epichlorohydrin

Ethylene-co-glycol (ECH) is a common cross-linker used in different types of biopolymers, including cellulose, starch, and others. An odorless, colorless, mildly dissolved in water but undissolved in a polar solvent solution with a low-molecular weight is produced when ECH is used to make hydrogels [115,116]. Gelation occurs at the very end of the polymer chain because ECH has a reaction with the hydroxyl group that is present in every polysaccharide. As a result of ECH, the pore size distribution, chemical stability, mechanical resistance, and adsorption/desorption capacity of a material can all be enhanced [117]. Increased water-holding capacity in the hydrogel network is a result of the addition of ECH as a cross-linker, which also increases pore size and pore formation in the hydrogel. However, the use of ECH in the preparation of chitosan hydrogels inhibits chitosan dissolving throughout heavy metal adsorption under acidic conditions and enhances metal adsorption capacities. As a result, epichlorohydrin induces phase separation and the formation of a heterogeneous network, which increases the hydrogel water absorption capacity [118]. The different concentrations of crosslinking agents in the solution cause phase separation. Rapid water diffusion in the hydrogel network is caused by a high ECH concentration, resulting in a large water absorption capability. The crosslinking chemical bond increases the water-absorption capacities as the amount of crosslinker increases. 

#### 4.2.3. Glutaraldehyde

Due to its low toxic effects, sufficient crosslinking capacity, high reactivity, relatively inexpensive, and ease of processing [119], glutaraldehyde is prevalently used as a crosslinking agent. It has been found that crosslinking glutaraldehyde with the hydrogen group is incredibly effective in developing functional polymeric materials from protein, amino polysaccharides, and synthetic polymers [120,121]. Instead of hydrogels, glutaraldehyde can be used as a ligands-modifier to remove heavy metal ions. In order to eliminate metal ions and increase the film’s water absorption, it is combined with chitosan as a crosslinking agent for modifying ligands efficiency [122,123]. Optometric drug delivery is another area where it is being studied. It was used as a chemical crosslinking agent in the synthesis of carboxyl-methyl chitosan hydrogels, producing hydrogels with a unique combination of properties. Hydrogels improve their bioactivity, swelling behavior, pH sensitivity, and rheological properties after gelation [124].

#### 4.2.4. Chemical Reaction of Complementary Groups

Water-soluble polymers have solubility due to the presence of functional groups (mostly OH, COOH, and NH_2_) that can be exploited to make hydrogels. Covalent linkages can be formed between polymer chains through the interaction of functional groups with complementary reactivity, such as an amine-carboxylic acid or isocyanate-OH/NH_2_ reaction, or through Schiff base formation. In addition, it has been reported that the chemical hydrogels can be cross-linked in a number of ways, including by condensation reactions, addition reactions, high-energy irradiation, and the use of enzymes.

#### 4.2.5. Enzyme Mediated Crosslinking

Enzymatic crosslinking has been utilized successfully to make polysaccharide-based hydrogels under controlled reaction conditions [125,126]. However, the implementation of the enzymatic crosslinking approach is constrained by its high cost and substrate specificity. Through the breakdown of hydrogen peroxide, horseradish peroxidase (HRP) can accelerate the coupling of phenol or aniline derivatives [127]. The acyl-transfer interaction between the γ-carbonyl group of a glutamine residue and the ε-amino group of a lysine residue is catalyzed by microbial transglutaminase (MTGase) [126]. To satisfy the substrate requirements of various enzymes, polysaccharides must first be changed. Therefore, it is difficult to achieve the performance requirements for wound dressings with a single crosslinking strategy. Combining the use of two or more different crosslinking techniques may result in additive benefits. For instance, chemical and physical links may be allocated to a single hydrogel, with the chemical linkages being in charge of the hydrogel’s stiffness and the physical linkages being in charge of its toughness. Exact management is necessary to maintain a delicate equilibrium between each interaction. Double-network hydrogels, made of two different kinds of polymer components with opposing physical natures, have been proposed in recent years [128,129]. Cross-linked rigid skeletons serve as the first network’s minor components, whereas poorly cross-linked ductile materials serve as the second network’s significant components [130]. Hydrogels may now operate in a variety of ways while also being mechanically durable due to new techniques.

#### 4.2.6. Disulfide Bonds

Incorporating dynamic disulfide groups into the main chains of cellulose is an additional efficient method for producing reversibly cross-linked cellulose-based hydrogels [131,132,133]. The disulfide bonds are reversible covalent bonds based on thiol/disulfide dynamic exchange reactions and are sensitive to pH or redox potential [134], providing a new physiologically compatible strategy for preparing dissociable materials hydrogels and micelles for drug and gene delivery [135,136].

Tan et al. [131] synthesized thiolated hydroxypropyl cellulose derivatives (HPC-SH) in 2011 without destroying the thermosensitive property of HPC. The cellulose nanogels were produced by the self-association of HPC-SH in solution at 45 °C, followed by the oxidation of thiol groups to disulfide bonds, which stabilized the associated structure. In this method, neither monomer nor cross-linker was used to prepare HPC nanogels, and the resulting nanogels exhibited both thermo- and redox-sensitive properties. The substitution degree of thiol groups (-SH) in the thiolated HPC could control the degree of cross-linking of the nanogels. The hydrodynamic radius of the nanogels can be adjusted by varying the degree of cross-linking, concentration of HPC-SH, and temperature. The dual stimuli-sensitive nanogels could find use in controlled drug release, transfer switch devices, and sensors. Following that, Hou and colleagues [133] demonstrated a novel pH and redox dual-responsive cellulose-based nanogel and used controlled release of agrochemicals. To facilitate the cross-linking reaction, aldehyde groups were grafted onto hydrophobic carboxymethyl cellulose (HCMC) through the addition of glyoxal. The obtained product (HCMC-a) was combined with solutions of salicylic acid (SA) and 3,3′-dithiobis (propionohydrazide) (DTP) to form a dual-responsive nanogel that exhibited pH and glutathione (GSH)-triggered release behaviors of SA.

In the same system, Liu et al. [137] created a cellulose-based multi-responsive hydrogel with enamine and disulfide bonds. The cellulose hydrogel was created by simply mixing at room temperature aqueous solutions of cellulose acetoacetate (CAA) and cystamine dihydrochloride (CYS). Because it contained a pH-responsive enamine moiety and a redox-active disulfide moiety, the cellulose-based hydrogel demonstrated dual-responsive properties with tunable release in response to pH and dithiothreitol (DTT) concentration changes.

Growing interest has also been shown in the structurally dynamic disulfide bond for the design of reversible bonding adhesive hydrogel. Cudjoe et al. [132] recently reported a strong, rebondable, semicrystalline disulfide nanocomposite network in which the thiol-endcapped polymer was dynamically cross-linked with thiol-functionalized CNCs via disulfide bonds. Due to the melting of the semicrystalline phase and the induction of the dynamic behavior of the disulfide bonds, increasing the temperature from 80 to 150 °C resulted in the rebonding of the nanocomposites with minimal loss in adhesive shear strength during rebonding.

### 4.3. Polymerization Method

Polymerization is another process for crosslinking in the production of hydrogels. Polymerization can be classified into three types: bulk polymerization, solution copolymerization, and irradiation polymerization. In bulk polymerization, only monomers and monomer-soluble initiators are utilized, and the concentration of the monomer affects the rate and degree of polymerization. Bulk polymerization has been shown to make hydrogel with a glassy, stiff, transparent hydrogel matrix [138,139].

In copolymerization, two types of polymerizations must be hydrophilic and arranged in a random, structured, block, or alternating network polymer configuration. Additionally, when the co-polymeric block is used in situ, it can form a hydrogel, which proves that the hydrogel is biodegradable and compatible with the body. Copolymerization makes hydrogel, which is usually used as a slow-drug release because it can release both drugs that do not mix with water and drugs that do. Co-polymerization can also be used to encase cells and repair damaged tissues [140].

According to Zainal et al. [62], synthetic polymers are often produced using irradiation polymerization. Its quick gelation period and the interaction of hydrophilic synthetic polymers and biopolymers with reactive groups result in the formation of macromolecule monomers. Hydrogels produced by irradiation polymerization could also be used in chemical applications. Thermo-responsive hydrogels are another type of hydrogels that are produced via irradiation-induced polymerization. Thermo-responsive hydrogels are widely used in drug release and cell adhesion. Radiation-polymerized thermo-responsive hydrogels exhibit great degradability under alkaline conditions and can be converted into oligomers for cell adhesion.

This has led to the acceptance of polymerization-based physical crosslinking as a standard procedure for making hydrogels. For the more challenging procedure, polymerization could be a widely employed method that aids in both production time and quality. In addition, hydrogel manufacturing relies heavily on crosslinking to preserve the 3D polymer network structure, which can be either chemical or physical [141].

## 5. Cellulose Derivatives

### 5.1. Hydroxypropyl Methylcellulose (HPMC)

The cellulose derivative HPMC is utilized a lot in controlled release applications because of its ability to thicken, gel, and swell. Additionally, it is safe to be using, easy to compress, has properties that make it swell, and can handle high drug levels. Due to its excellent bioactivity, HPMC can be a thermo-sensitive natural polymer that forms a transparent, highly stable colorless hydrogel, rheological properties, and changes in texture. Gårdebjer et al. [142] investigate the pore-forming effects of hydroxypropyl methylcellulose mostly in MFC (micro-fibrillated cellulose) film and adjust the wettability characteristics of the films. The results demonstrate that HPMC can have a potent reaction with MFC films where it might create h-bonds at the surface of the film.

Hydroxypropyl methylcellulose, being used in scaffold engineering, was created from cross-linked chitosan by Hu et al. [143]. After promoting cellular characteristics, they demonstrate that crosslinking hydroxypropyl methylcellulose with chitosan can give the recovery process structural strength and shape. The use of HPMC as a composite hydrogel in scaffold engineering was also maintained by Yin et al. [144]. This research implies that the HPMC composite hydrogel can facilitate faster healing, a more uniform distribution of cells, and a reduced risk of complications during osteoplasty procedures. Hydrogel scaffolds, films, and membranes are typical applications for HPMC in the sector of medicine.

### 5.2. Ethyl Cellulose (EC)

The glucose units in cellulose are changed into the ethyl ether groups to produce EC, which is a biopolymer. The EC of this polymer is unaffected by the pH of the environment. It does not dissipate in water because it is non-ionic, but it does dissipate in solvents that are polar. This polymer functions as an insoluble component in matrix or coating strategies and as a non-swelling polymer [145]. Because the active ingredient is sensitive to water, EC is used in dosage processing when water-soluble binders cannot be employed.

It is possible to coat the tablets with this polymer so they will not react with another substance. This polymer may also be utilized in combined application with other polymers to protect against the discoloration of easily oxidized materials, such as vitamin C. In order to create a sustained-release film coating for coating tablets, pellets, and microparticles, this polymer was mixed with water-soluble polymers [146].

### 5.3. Carboxymethyl Cellulose (CMC)

CMC is a water-soluble derivative of cellulose that is widely used in the biopolymer industry. It is made when 2, 3, and 6 of the hydroxyls on the backbone of cellulose are replaced by carboxymethyl groups [147]. Cellulose containing numerous hydroxyl groups is an abundant and inexpensive natural biopolymer, making it an attractive starting material. In addition, CMC possesses bioactivity, solubility, and bio-degradability. CMC is prepared in a non-aqueous monochloroacetic acid/soda solvent medium in order to reach the substitution degree via carboxymethylation [62]. Hydrogel composed of CMC has potential applications in enzyme immobilization, wound healing, drug delivery, and adsorption. Hydrogels composed of nanoparticles/CMC can be utilized for antimicrobial properties, wound healing, drug development, and tissue formation. The nanoparticles added to carboxymethyl cellulose hydrogel improve the hydrogel’s performance. 

Nanoparticles contribute to the enhancement of carboxymethyl cellulose hydrogels through their superior mechanical, electronic, optical, and physicochemical characteristics. Carboxymethyl cellulose derived from pineapple plants serves as an efficient carrier for papain immobilization and forms a strong H-bond between the employed materials. Even though CMC can be easily extracted from biomass resources, bagasse and empty fruit bunch were also used to produce carboxymethyl cellulose. Every type of biomass resource imparts unique characteristics to CMC, such as exceptional absorption and adsorption, a high swelling ability, and superior optical properties. In addition to being advantageous for the production of CMC hydrogels, the high level of methylation group in various biomass wastes is also beneficial.

### 5.4. Nanocellulose (NC)

With a density of 1.6 g/cm^3^, a low molecular weight, a large surface area, stiffness up to 220 Gpa elastic modulus, and strength up to 10 Gpa TS, nano-cellulose, which is produced from pure cellulose at the nanoscale, possesses a number of amazing qualities [148]. Cellulose nanocrystals (CNC), nano fibrillated cellulose (NFC), and bacterial nano-cellulose (BCNC) are three styles of nano-cellulose with comparable chemical components but distinct morphologies [149]. CNC is created by the acid hydrolysis of cellulose nano-fibrils, additionally referred to as 100 percent cellulose nano-whiskers. 

NFC is likewise referred to as cellulose nano-fibers, nano-fibrils, nano-cellulose fibrils, cellulose micro-fibrils, and micro-fabricated cellulose. NFC is adaptable, has a prolonged, tangled configuration, and has a radius of 1 to 100 nm and a length of 500 to 2000 nm [150]. NFC is made up of 100% cellulose, which may be either crystalline or crystalline [151]. NFC is bigger than CNC in phrases of floor location, element ratio, and duration. The size of BCNC is 20–100 nm [152], and it looks like a twisted ribbon. *Gluconacetobacterxylinus* is a bacterial species that produces lower molecular weight sugars that are commonly used to make [153]. In current years, nano-cellulose has been used a lot to make hydrogels for many different uses, particularly in the food packaging industry.

### 5.5. Cellulose Nitrate (CN)

Nitrocellulose, also known as CN or gun cotton, is a key component of smokeless gunpowder due to its propensity to decompose explosively. By nitrating cellulose obtained from wood or cotton linter pulp using potent nitrating chemicals, like nitric acid, CN is created. Because of the electrophilic outburst of NO_2_^+^ ions on the OH moieties during the nitration process, the hydroxyl groups on the surface of cellulose are transformed with nitrate esters [154]. This mechanism was initially studied in the presence of alcohol and amine nitration, and further research has demonstrated that it applies to cellulose as well. A complete nitration of cellulose is characterized by a final nitrogen concentration of 13.5% or more in CN [155]. The applications of CN depend significantly on the nitrogen content. CN containing between 12.6% and 13.3% nitrogen is categorized as an explosive and utilized as a gun propellant, but CN containing less than 12.6% nitrogen possesses high biocompatibility and physicochemical stability and is deemed suitable for biomedical uses. Non-covalent interactions between the nitro groups of the polymer and the amine functional groups in the protein structure make CN membranes useful for protein preservation. In addition to their use in biosensors and hemodialysis, this quality makes them a versatile material. Furthermore, CN is used in electrophoresis films, osmosis membranes, and ultrafiltration membranes [154].

### 5.6. Cellulose Sulphate (CS)

Cellulose sulphate (CS) is an ester of cellulose produced using homogeneous, quasi-homogeneous, and heterogeneous sulfation techniques. CS possesses high levels of water solubility, a high substitution degree, and antibacterial properties. When the sulfonic acid groups are raised from 528 to 689 µmol/g, CS is easily soluble in water, resulting in a translucent solution [156]. In the transitional reaction of CS, cellulose nitrate is produced. The sulfation was accomplished by dissolving cellulose in N, N-dimethylformamide with a mixture of sulfating agent (chlorosulfuric acid) and acetylating agent (acetic anhydride), then cleaving the acetyl group and converting cellulose acetate-sulfate into CS upon precipitation. This is known as acetosulfation or a quasi-homogeneous process.

The sulfation was accomplished based on the hydroxyl groups of anhydrous glucose unit C_2_ and C_3_ sites; for instance, the presence of hydroxyl groups in C_2_ and C_3_ positions suggests acetosulfation, whereas the absence of hydroxyl groups in C_2_ and C_3_ positions shows homogeneous sulfation. Therefore, the biological characteristics of CS depend mostly on the degree of cellulose interconversion and its molecular weight [154]. Recently, Christian Willems et al. created bioactive hydrogel by mixing oxidized CS with cross-linked carboxymethyl chitosan [157]. Based on molecular weight and degree of oxidation, they found that the hydrogels are biocompatible against cytotoxicity for 14 days with live cells. This hydrogel can be utilized in tissue engineering as a replacement to several types of connective tissue. The oxidized CS and carboxymethyl chitosan-based hydrogels have a greater G′ value as the molecular weight, crosslink density, mixing ratio, and time are increased towards the maximum number of crosslinking that can form in the hydrogels. The G′ value explains the gelation property of hydrogel, with a higher G′ value indicating greater gelation [158].

Gelation is a very important process that turns a liquid into a solid. This process makes it possible to make a wide range of foods with different textures. Ting et al. made microcapsule hydrogels with polyphosphates patched onto CS-chitosan hydrochloride to deliver 5-aminosalicylic acid, an anti-inflammatory drug used to treat ulcerative colitis and Crohn’s disease [159]. The microcapsules are good drug delivery vehicles because they can hold about 66.9% and 4.6% of the drug and encapsulate it well. The structure and properties of microcapsules are similar to those of sago, which is made from starch and is often used in porridge and cold coffee. In the same way, the microcapsules could also be used to deliver bioactive compounds such as vitamins, minerals, natural flavors, antioxidants, antimicrobials, and other compounds that help treat diseases in human diseases.

### 5.7. Cellulose Acetate (CA)

Acetic anhydride is used to create cellulose acetate (CA) from wood pulp. CA is also generated from cotton through a reaction of sulfuric acid and acetic acid. Acid hydrolysis can be used to convert the resulting cellulose triacetate into cellulose diacetate and CA. The degree of substitution in cellulose ester is critical for its solubility and biodegradability. CA is insoluble in water and has excellent mechanical qualities, low water content, and low swelling. CA’s hydrophilic nature and water solubility are mostly due to its hydroxyl groups [154]. The CA is employed as a membrane in a variety of applications, particularly biomedical fields, for separation, adsorption, biosensing, drug administration, catalysis, and tissue engineering. Electrospinning, progressive electrostatic assembly, phase inversion by solvent evaporation, and immersion precipitation are the most often utilized manufacturing procedures for CA synthesis. CA fibers created by electrospinning offer a wide range of uses, including packaging, hydrogels, sensor composites, fiber films for wound healing mats, medication delivery, scaffolds in tissue engineering, protein control membranes, and biosensors.

CA with a degree of substitution (DS) of 2.5 is used to make the hydrogel, which is then combined with ethylenediaminetetraacetic dianhydride (EDTAD) as a crosslinking agent. Triethylamine is added after complete mixing to improve viscosity and act as an esterification catalyst in hydrogel production. For 5–10 min, the water absorption capacity was determined to be 550% at 25 °C and 1000% at 50 °C [160]. Furthermore, via esterification crosslinking and using triethylamine as a catalyst, the hydrogel from CA (DS 2.5) with EDTAD was used as a reduction substrate of NPK fertilizer in soil. The created hydrogel works well as a substrate for slow release, water retention, and fertilizer leaching reduction. Due to its high-water retention capacity, non-toxic, biodegradable, and environmentally friendly nature, this can be utilized in drought-prone locations where water availability is limited for agriculture and horticultural production [161].

Regenerated cellulose nanofibers derived from deacetylated electrospun CA nanofibers demonstrated greater compressive strength, water imbibition, increased biomineralization, and improved pre-osteoblast cell survival, adhesion, and proliferation. This study shown that CA-based hydrogels can be effective 3D bio-scaffolds for bone tissue engineering [162]. The study was focused on biomedical and food applications. As a result, CA-based hydrogels may have use in food packaging as humidity absorbers in high moisture foods, extending the shelf-life of packed meals. More research is needed to build CA-based functional hydrogels with the addition of diverse bioactive chemicals that have good impacts on human health.

## 6. Potential Applications in the Food Industry

**Figure 4:**
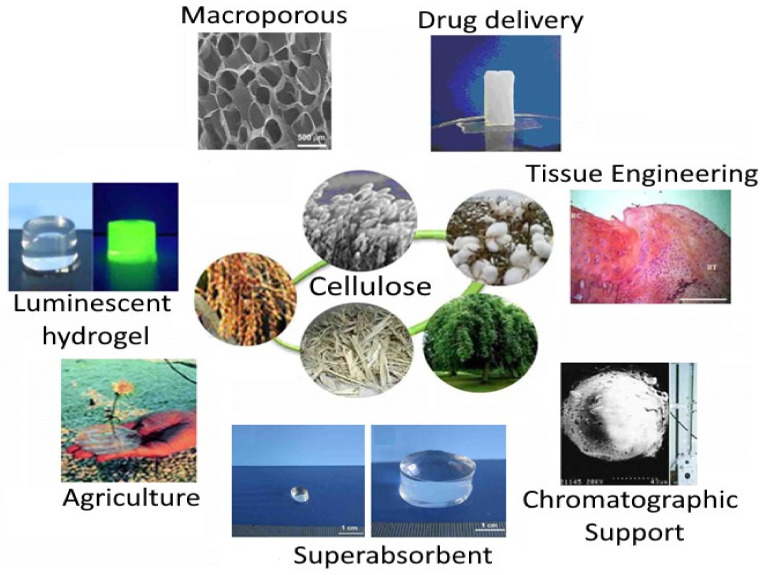
Applications of cellulose-based hydrogels (CBHs) in different fields. Figure 4 is adapted with permission (Copyright © 2010 Elsevier Ltd., Amsterdam, The Netherlands) from Chang and Zhang [163].

Hydrogels are utmost widely utilized polymer classes due to their versatility in a wide variety of applications across the biomedicine. These include contact lens material, bone marrow cartilage, and pharmaceutical and cosmetics industries. The popularity of hydrogels in the field of food processing continues to increase annually at the academic level, but not in the commercial sector due to less knowledge and understanding on the part of the public zone. This has prompted researchers to concentrate on developing and eventually marketing hydrogel-based food processing products for use in the food manufacturing industries (Figure 4).

### 6.1. Food Biosensors

Nutrition and safety are crucial in the food processing sector. Conventional analysis methods are repetitive, time-consuming, and require experienced workers, necessitating rapid, efficient food standards and quality control. Biosensors replace conventional methods; because of their velocity, ease of mass fabrication, precision, field applicability and economics, and biosensors are becoming important in the agricultural and food industries. The gadgets incorporate a transducer and an immune response, organelle, enzyme, or microorganism. Organic compound interacts with analytes, and transducers convert organic responses to electric signs. Biosensors detect the fermented carbohydrates, alcohol, and acids. 

A hydrogel biosensor is a rapid, inexpensive, and non-destructive method to assess the food’s quality. A functional hydrogel consisting of silver ions, D-glucose pentaacetate, and agarose is utilized to evaluate the growth of biogenic amines (BAs), which indicate the freshness of fish. Unlike other BAs sensors that have been reported before, this hydrogel-based biosensor does not need to make a fluorescence probe first. This makes it cheaper and easier to use for monitoring the cleanliness of fish. The hydrogel is also used to test bacterial trapping and toxicity assessments.

CNC is employed in biosensors due to its structure resembling biological tissue, viscoelasticity, biocompatibility, and self-healing. CNC-based hydrogels can identify pH changes in food products due to their surface-bound fluorophores and hydroxyl groups. CNC-based hydrogel can detect toxic compounds and pesticide residues in food. The biosensor made from CA/CNTs/cholesterol oxidase demonstrated superior performance and high accuracy with a limit of detection of 108 M. In another study, a biosensor made of cotton N,N-dimethylacetamide/cellulose/Titanium dioxide/lithium chloride nanoparticles measured glucose. Physical adsorption links the glucose oxidase enzyme to Titanium dioxide in the nanocomposites biosensor. The glucose biosensor was linear from 1–10 mM. As a biosensor for food quality, hydrogel is being researched. The evaluation suggested that research be done in this field because of its quick response time, cost-effectiveness, and biodegradability.

### 6.2. Hydrogels Based on Cellulose for the Industry of Food Processing

Utilizing hydrogel as a component of a smart packaging system or as a carrier system that is successfully incorporated into food items are a few examples of potential applications in the food industry. Their principal purpose as a component of a smart packaging system is to communicate information regarding the freshness of the fresh food contained inside or as a quick testing method for determining the presence of dangerous compounds such as aflatoxin. Other innovations of hydrogel also provide their use as carriers of flavors or biologically active compounds such as carotenes, which are typically implemented in nano-emulsions.

### 6.3. Food Packaging Industry

CBHs have the potential to be utilized in food packaging systems. Recent attention has been generated by moisture absorption techniques for processed meals that rely on “absorbent pads” with water-removal capabilities because they may lower the danger of microbial contamination while keeping the sensuous qualities of packaged food [164]. This active food packaging can serve multiple purposes, including the absorption of food-derived fluids, reconfiguration of the packaging headspace, and anti-microbial properties.

Most commonly, absorbent materials are used in food packaging systems in which a plastic tray or container is used to collect liquids released from food while it is being stored. Hydrogels can be used to control the water activity of food products while also absorbing any exudates that might be released during packaging.

There are four guidelines that must be fulfilled when using absorbent materials in food packaging systems. The absorbent material must satisfy several criteria: (i) after absorption, it must maintain the exudate in the 3-dimensional formation; (ii) the packaged food’s good aesthetic presentation and sensory qualities must be preserved using absorbent materials at a fair price; (iii) the absorbent material must exhibit specific performance qualities to guarantee the framework’s structural integrity throughout storage; and (iv) the absorbent material must lengthen the storability of storage food product [165,166].

Hydrogels derived from cellulose can be employed as energetic packaging and moisture content regulators in packaged meats, fruits, vegetables, and other products with high water content [167]. Another study found that using lactic and acetic acids to clean meat are healthful. Due to their low cost and ease of use, solid anti-microbial are widely used in the food manufacturing business. Enzymatic crosslinking of cellulose and its derivatives with lactic and acetic acids creates anti-microbial hydrogels. In comparison to *Listeria monocytogenes*, *Escherichia coli* was more resistant to cellulose-based hydrogel films containing ZnO, CuO, and AgNPs. Cellulose-based hydrogel film can extend fresh potato storability [168]. Raw potatoes are wrapped and placed in plastic boxes because their higher rate of respiration throughout the storage causes fogging. CBH films’ antioxidants (ferulic acid) prevented butter lipid oxidation. The literature identifies that CBHs are important in active and intelligent food packaging. Hydrogels’ active compounds protect food from deteriorative reactions, increasing shelf life and quality.

### 6.4. Hydrogels Derived from Cellulose for Use in Healthy Foods

#### 6.4.1. Enzyme Immobilization

Enzymes are unsteady, non-reusable, and have a short lifespan, which is important in food processing. Enzyme immobilization improves the stability, reusability, and longevity of biocatalysts utilizing various carriers. An immobilized cellulose-based hydrogel with enological Pectinase can be used as a biocatalyst in wine production because it reduces grape juice turbidity at 25 °C for 160 min. This biocatalyst can be excluded and reused after clarification, reducing wine manufacturing costs [169]. Immobilized hydrogels containing pectinase are used to clarify fruit juice. For those who are lactose intolerant, enzyme immobilized hydrogel is used to create low-dose or lactose-free milk. With hydrolysis of regular lactose and UHT milk lactose, immobilized lactase activity varied between 95.92–55.03% and 95.92–72.85% from 0 to 10 cycles, respectively. To make lactose-free milk, use hydrogel-immobilized lactase for 10 hydrolysis cycles [170,171].

According to the findings of some studies, CBH-fortified hydrogel has potential as a functional ingredient. Due to oral mucosa absorption and slow-release properties, functional hydrogels can deliver the target nutrient, increase its bioavailability, and better human health. Jell-O is a gelatin, sweetener, artificial color, and flavor hydrogel product. This product helps develop functional hydrogels. It is made from synthetic chemicals that can harm the body if consumed excessively or regularly. 

#### 6.4.2. Encapsulation

Many diseases can be cured via encapsulation, which protects and delivers bioactive molecules to their target areas. It also meets the growing demand for nutritious and tasty foods [172]. During microencapsulating of CBHs, primary, secondary, and tertiary parameters should be considered. The main determinants are the choice of biopolymer, molecular production techniques, and material characteristics including polarity, charge, and environmental sensitivity. Secondary factors include sensory characteristics (flavor, appearance, texture), storage conditions (temperature, ionic strength, aw, pH, and mechanical stresses), and shelf-life. Tertiary factors determine the release location, profile, and conditions. The process of encapsulating active ingredients improves the solubility or dispersion of the ingredient in the food, as well as the ability to mask the taste, maintain storage stability, and control the release of the ingredient. CBHs can protect probiotic cells in a capsule from the harmful environment, thus extending their life. Hydrogels help probiotic microorganisms live in the intestines and at different temperatures. According to the study, enough live cells can endure handling, storing, and digestion in order to make it to the gut unharmed [173].

### 6.5. Texture and Disease Control

A significant number of studies are investigating the possibility that hydrogel particles could be incorporated into food in order to impart a different consistency. Cellulose based hydrogel microspheres can be utilized in place of fat (starch granules and fat droplets) to make foods lower in calories. Hydrogel particles have the same microstructure and rheological characteristics as swollen starch granules, such as high yield stress and high shear viscosity. Additionally, the way cellulose melts, absorbs oil, and stays stable at high temperatures are important parts of fat-rich foods. When CBHs are made stronger with gelatin, they can be as reliable as fat droplets in the mouth [174].

CBHs enhance the biologically active substances that will aid in the treatment of a variety of health disorders by incorporating bioactive compounds into food systems such as fluid gel (sauces, smooth cream, and beverages), soft gel (heavy cream, ghee, and chocolate cake), and hard gel (cheesecake) (sauce, ketchup, and candies) [175]. Silver, copper, titanium, and zinc nanoparticles are the most widely used, and they exhibit remarkable antimicrobial properties. Liver damage and cancer are facilitated by nanoparticles, which can be easily absorbed into the blood after migrating from food packaging. Therefore, cellulose encapsulation of nanoparticles to prevent nanoparticles migration is a potential solution to this issue [176].

### 6.6. Food Preservation

Several factors have been identified as causing post-harvest deterioration and damage to fruits and vegetables in studies. Climate and weather conditions, such as heavy rain and high winds that cause harvesting damage, have the significant influence on the quality of fruits and vegetables. Inadequate fruit and vegetable handling, including the use of ineffective equipment and the absence of improved materials and equipment with the passage of time, transit, and storage, is also a significant issue. If enough care is not exercised in packaging and marketing scenarios, post-harvest losses may occur. Other factors, such as bacteria, bruising and cuts, bugs and rats, birds and other animals, inadequate packaging and low-quality storage buckets, and the use of wooden boxes, all contribute to the rapid deterioration of fruits and vegetables. To address the bulk of these concerns, cellulose and its derivatives are used in packaging. Hydrogels are used in the food industry to improve the stability and bioavailability of bioactive food ingredients [177]. Edible hydrogels can also be used to extend the shelf life and quality of products by encapsulating active ingredients such as antimicrobials and antioxidants [178]. Chen et al. [179] examined the use of food proteins as substrates in nutraceutical hydrogel delivery systems. The hydrogels formed provided biocompatible carriers for the oral administration of sensitive nutraceuticals in a variety of food products. Biodegradable food packaging is another common application for hydrogels in the food industry [180]. 

Edible coatings offer a cost-effective and ecologically responsible way to improve food quality and extend food preservation during refrigerated storage. Coatings can be made from a variety of materials, including carbohydrates (starch, cellulose, alginates), proteins (gelatin, whey protein, casein, and zein), and lipids (waxes, oils, fats) [181]. Hydrogels, oleogels, and bigels were used as coatings on fresh meat and fishery products. As a result, edible coatings could be made from systems such as hydrogels, oleogels, or a mixture of the two, known as bigels [182]. The meal is directly immersed in a liquid solution during the coating process. Edible coatings can act as a barrier to oxygen and water ingress in food, reducing oxidation reactions and maintaining moisture. Various edible coatings, such as chitosan coatings on Indian oil sardines (*Sardinella longiceps*) [183], chitosan-gelatin coatings on shrimp (*Litopenaeusvannamei*) [184], and sodium alginate or whey protein coatings on rainbow trout (*Oncorhynchus mykiss*) fillets [185] have been studied for the preservation of fishery products during refrigerated storage.

Hydrogels are three-dimensional, hydrophilic macromolecular networks that retain a considerable amount of water due to interactions between the polymeric chains of a gelling agent [186]. Furthermore, most hydrogels are reversible, with the ability to change their rheological characteristics in response to changes in external circumstances (temperature, pH, ionic solution strength, etc.) [187]. Because of its gelling properties and resistance to dehydration, light, and oxygen, gelatin is an excellent coating material.

Oleogels are three-dimensional, anhydrous, viscoelastic gels that are formed when low molecular weight or polymeric structures are added to edible oils, which cause the continuous phase of the solution to become structured [188]. Low molecular weight oleogelators include waxes, fatty acids and alcohols, lecithin, monoglycerides (MGs), and a combination of phytosterols with oryzanol [189] or MGs [190]. Structured oil has been shown in studies to effectively replace animal fat in foods [191,192,193,194]. The potential for oleogel and oleogel-based devices as delivery platforms for lipophilic bioactive chemicals is enormous [195].

Bigels (hybrid gels) are biphasic systems in which the lipid and aqueous phases, respectively, are organized as oleogels and hydrogels [196,197]. Technically, bigels resemble emulsions with a gel network in both their aqueous and lipid phases, but they have superior physicochemical stability over time compared to simple emulsions [197]. Bigels are formed by dispersing one phase into the other, with oleogel-in-hydrogel bigel systems being the majority [198]. Bigels are advantageous for the regulated distribution of both hydrophilic and lipophilic bioactive compounds due to their two structural phases [199]. In addition, their relatively simple manufacture [200], spreadability [199], longer shelf life [200], and stability for 6 to 12 months at ambient temperature [201] make these systems suitable for use as edible coatings for meals. Some food-grade bigels are currently used as possible fat alternatives in food products [202,203].

#### 6.6.1. Fruits Preservation

Fruits are perishable and regularly consumed. Fruits are an essential part of our diet since they are rich in necessary nutrients, vitamins, and minerals, all of which contribute to a balanced diet. The high sugar and water content in fruits creates an ideal home for microorganisms. Ethylene is a natural chemical produced by ripening fruits and responsible for their decomposition [204]. Cellulose containing methylcyclopropene has proven to be a significant and effective therapy for ethylene production prevention [205]. The invasion of microorganisms is another way spoiling can develop. The majority of fruit deterioration is caused by microorganisms. Cellulose is actively utilized to include diverse antimicrobial compounds in order to ensure their continuous release from the matrix following activation. A wide variety of factors can contribute to spoilage, including but not limited to: ethylene gas, microbial spoilage, improper storage conditions, rodents and insects, poor grading during harvest and storage, moisture in storage areas, improper cutting or harvesting techniques, and contaminated packaging. To improve this, these fruits are packaged with a variety of packaging materials. Water loss, surface dehydration, translucency, softening, browning, germs, texture loss, off flavor, and disagreeable odor are the primary reasons of the deterioration of freshly cut fruits and vegetables. Moisture-absorbent pads used in trays of freshly cut fruits and vegetables can also contribute to food spoilage by serving as a breeding ground for numerous types of bacteria, hence causing food waste [206]. All of these problems can be solved by using materials for food packaging that is safe, natural, and compatible, for example, cellulose. 

#### 6.6.2. Vegetable’s Preservation

Freshly cut vegetables are gaining popularity because they are simple to prepare and save a great deal of time. The weakening of freshly cut vegetables is one of the most significant problems associated with freshly cut foods. This may be owing to the chopping, dicing, and slicing techniques needed to prepare vegetables, which cause them to lose their firmness. As fibers are slashed, juices seep out, leading in the softening of food, particularly vegetable tables [206]. This could result in texture loss, which is unacceptable to consumers. Modified environment packaging does not aid in texture preservation. According to scientists, edible wrapping is one of the most practical ways to avoid the softening of vegetables. Consequently, cellulose is among the utmost significant of edible covering materials and is commonly used in packaging [207]. Calcium chloride and other additives that improve the texture can be used with cellulose in a straightforward manner without having an effect on the substance’s qualities. The encapsulation and activation of naturally occurring antibacterial components are both helped along by cellulose. In foods such as tomatoes, one of the signs of spoilage is the emergence of translucency, which is characterized by the darkening of certain places that take on the appearance of bruises. This type of injury, which is known as chilling injury, is more common in colder regions. This is one of the most widely known problems associated with vegetables, and it makes the freezing preservation process challenging [206]. As a consequence of this, cellulose is used as a stabilizer, and it also includes a wide variety of preservatives and compounds that contribute to the solution of problems of this nature. The process of preserving vegetables by employing cellulose packaging is broken down in Table 2, along with the functioning of cellulose and the various films that can be used.

## 7. Conclusions

The hydrogel obtained from cellulose is inexpensive, readily accessible, simple to prepare, biodegradable, and possesses excellent functional characteristics. CBHs are widely used in wastewater treatment as adsorbents for toxic metals, dyes, and other substances. CBHs have also been proposed as biosensors for detecting adulterants and toxins in foods. CBHs are also used as functional foods, transporting nutrients and bioactive compounds that can help treat a variety of diseases. Due to their great degree of adaptability, simplicity of preparation, and biodegradability, CBHs are heavily researched in packaging solutions. Due to their flexibility, capacity to hold water, and response to stimuli, CBHs are utilized in packaged meals as smart packaging to observe the nutritional content of foods. However, the entire process and digestibility of hydrogels in vivo is still unknown and will require further research. The study revealed that the least expensive bioavailable CBHs can be used efficiently and successfully for a variety of food industry applications in this regard.

## Figures and Tables

**Table 1:** Materials, methods, and applications employed in the preparation of hydrogels.

Materials	Methods	Applications	References
Polyacryl amide Hydroxyethyl cellulose	Radiation-induced Chemical crosslinking	Agricultural product processing Wound dressing	[48] [49]
	Free-radical polymerization	Self-healing	[50]
	Grafting	Bacteriostasis	[51]
Carboxymethyl cellulose	Freeze–thaw Layer-by-layer assembly (Fabrication method)	Enzyme immobilization Improved preservation of beef	[52] [53]
	Gamma radiation	Hemostat hydrogel	[54]
	Grafting	Metal ions removal	[55]
	Co-polymerization	Pigment removal	[56]
	Chemical crosslinking	Drug carrier agent	[57]
	Chemical crosslinking	Anti-counterfeiting and labelling	[58]
Hydroxypropyl methylcellulose	Chemical crosslinking	Drug delivery	[59]
	Radiation	Scaffolds	[60]
	Chemical crosslinking	Controlled release	[61]
Hydroxypropyl cellulose	Pre-polymerization	Anti-fouling	[62]
	Freeze–thaw	Biomedical	[63]
	Chemical crosslinking	Thermoresponsive hydrogel	[64]
	Photo-crosslinking	Biomedical	[65]
Polyvinyl alcohol	Freeze–thaw	Biomedical	[66]
	Freeze–thaw	Regenerative medicines	[67]
	Freeze–thaw	Drug release	[68]
	Freeze–thaw Freeze–thaw	Radome materials Food packaging	[69] [70]
Polyethylene glycol	Gamma-radiation	Scaffolds	[71]
	Photo-polymerization	Implants	[72]
	Chemical crosslinking	Anti-biofilm activity and food packaging	[73]
Starch Cellulose nanofibril	Radical polymerization	Wound dressing	[74]
	Freeze–thaw Air-drying castingCombined with Polyvinyl alcohol and ascorbic acid	Biomedical Food packaging Food 3D-printing materials	[75] [76] [77]

**Table 2:** Cellulose and its derivatives used with the other polymers to preserve food.

Polymers	Role of Cellulose	Film’s Activity	References
Cellulose/silver nanoparticles	Add silver particles for antibacterial protection and to increase shelf life.	The film demonstrated significant antibacterial action against *Aeromonas hydrophila*	[208]
Bacterial cellulose	Cellulose was used to transport plant extracts and ensure their delayed release.	Prolonged shelf life and decreased post-harvest microbial storage	[209]
Cellulose/polylactide	Provide coatings with enhanced antioxidant characteristics.	Add enhances the flavor and freshness of tomatoes.	[210]
Cellulose and chitosan	Enhanced thermal and anti-bacterial attributes.	Prolonged shelf life for ground meat.	[211]
Carboxymethyl cellulose and chitosan	Increase chitosan solubility.	Enhance antimicrobial effect by adjusting chitosan concentrations.	[212]
Cellulose	Adhesive derived from cellulose for active packaging.	Increased cheese freshness and shelf life.	[213]
Nanocellulose	Nanocellulose has gas barrier qualities that limit leaf respiration.	A longer storage life and larger capacity for storing the product were achieved.	[214]
Cellulose nanocrystals/chitosan	Enhancement of mechanical and barrier characteristics.	Increased shelf life to 20 days.	[215]
Cellulose based on ethylene	Enhanced water vapor transmission and moisture absorption.	The trigger was the manufacture of ethylene.	[216]

## Data Availability

Data is contained within the article.
